# A case of early onset renal sarcoidosis complecated with multiple enchondromatosis

**DOI:** 10.1186/1546-0096-9-S1-P8

**Published:** 2011-09-14

**Authors:** U Kaneko, S Toyabe, M Uchiyama

**Affiliations:** 1 Department of Pediatrics, Niigata University Niigata, Japan

## Background

Early onset sarcoidosis (EOS) is a multisystem granulomatous disorder characterized by uveitis, arthritis, and skin rash with infiltration of noncaseating granuloma. Osseous and renal involvements are rare in EOS. Cyst-like radiolucencies are predominant radiographic pattern of osseous sarcoisosis, which mimic enchondroma.

## Aim

We present an unusual case of EOS with renal and osseous involvement. Histological findings of the kidney showed granulomatous interstitial nephritis, but bone biopsy indicated complication of enchondromatosis.

## Methods

A five month old boy suffered from skin rash on his whole body after vaccination of BCG. At three years old, he developed fever and painless soft mass on his right ankle. Enhanced abdominal CT scan showed multiple low density lesions in both kidneys. He was initially diagnosed as having acute focal bacterial nephritis. However, antibiotics were not effective and he was referred to our hospital.

## Results

Radiological findings of bone showed cyst-like radiolucencies in multiple regions such as phalanges of hand and feet, long bones, and pelvis. MRI showed tenosynovitis of ankle joint. Histological findings of skin, kidney, synovium and tendon sheath of ankle joint demonstrated the presence of non-caseating epithelioid granuloma. However, bone biopsy specimen revealed cartilage. He was diagnosed as having EOS complicated with multiple enchondromatosis. He was treated with oral corticosteroid at the dose of 1mg/kg, resulting in improvement of clinical symptoms.

## Conclusion

There are no signs and symptoms at the onset of renal and osseous sarcoidosis. We may have to carefully exam these involvements even in EOS. The complication of enchondromatosis needs further investigation.

